# A Centre of Veterinary Education

**Published:** 1898-05

**Authors:** 


### A CENTRE OF VETERINARY EDUCATION.

The Empire State dates the organization of one of the earliest and most successful veterinary colleges in America. This State has brought forth the largest number of graduates in veterinary science of any of the States. The progress of veterinary science in all its avenues of usefulness and the promotion of public welfare has been largely directed by graduates of her colleges. Veterinary sanitary work all over the land has felt the kindly influences of those who were graduated within her borders. To-day her position as a leading centre stands on very uncertain ground. Higher veterinary education within her border seems to have moved in recent years along uncertain and unwise lines, and they have brought some of her schools to face a condition that threatens their death, if not by a rapid process, by a decline in their value and usefulness that will make the more sudden demise much to be desired. Without support, without means, without such necessary aid in such work, there must naturally follow a decline in growth and development that will take to other fields the centre of veterinary education, and her rank will soon be wiped away. The reduction of the Board of Regents' requirements, now promised, will probably aid the schools in tiding over the situation of the past two years; but the question forces itself strongly upon all who have earnestly studied the situation: Is there room for two veterinary schools in New York City? All will unite in saying that for valuable clinical material it holds the highest place in our land, and this should not be lost sight of in the interests of those who are to fill the future ranks of the profession. The able teachers of these two schools could be united to form one of the best institutions of learning in the land, and were it associated with Columbia or some other university, it would give it a solid foundation, and this centre of veterinary education would be preserved for years to come. Who will move first in this much-to-be-desired end?

